# Neuroimaging studies of addiction: The need to incorporate real life data and profile heterogeneity

**DOI:** 10.1192/j.eurpsy.2023.37

**Published:** 2023-07-19

**Authors:** G. Sescousse

**Affiliations:** INSERM, Lyon Neuroscience Research Centre, Vinatier hospital, Lyon, France

## Abstract

**Abstract:**

Neuroimaging studies of addiction seek to understand the brain mechanisms that predispose to and support the maintenance of addictive behaviors. Traditional studies are *case-control cross-sectional* studies, i.e. they conceptualized individuals suffering from addiction as a homogenous group, and report lab-based experiments conducted at one particular point in time. In this talk, I will argue that a refined understanding of addictive behaviors requires the use of *dimensional longitudinal* studies. Using dimensions will reveal the existence of *heterogenous profiles* within diagnostic groups, and allow researchers to incorporate *individual variability* in their models. In turn, using longitudinal follow-up measures should allow researchers to determine whether brain-related abnormalities are *predictive* of symptoms in *real-life.* I will illustrate these points using a few example studies from the literature.

**Disclosure of Interest:**

None Declared

